# A Unique Metro Choice Behaviour of Suburban Rail Passengers in India

**DOI:** 10.1007/s40864-022-00184-9

**Published:** 2023-02-27

**Authors:** M. Selvakumar, D. Siddi Ramulu, K. Sankar

**Affiliations:** 1 Department of Civil Engineering, Sri Venkateswara College of Engineering (SVCE), Sriperumbudur, Tamil Nadu 602117 India; 2 L&T Infra Engineering Limited, TC 2 Third Floor, Mount Poonamallee Road, Post Box No: 979, Manapakkam, Chennai, Tamil Nadu 600089 India

**Keywords:** Modal shift, Metro, Logit, Stated preference survey, India

## Abstract

This study aims to analyse the inter-rail modal shift behaviour of suburban rail passengers to examine ridership for the proposed metro extension corridor in Chennai, India. This investigation was conducted in 2019 as part of a feasibility study for the extension of the metro line spanning between Chennai Airport and Kilambakkam, a southern suburb of Chennai. The same origin–destination pair is also served by the suburban rail system. It is an extension of the operating line from Washermenpet to the airport of the Phase I metro project. For this inter-rail competition study, a sample of 272 suburban rail passengers covering work, education and other trip purposes were interviewed using a stated preference questionnaire. Six stated scenarios were considered for analyses which included travel time saving by using the metro along with the fare difference between metro and bus. The study revealed that suburban rail passengers were less concerned about travel time saving and gave priority to fare difference irrespective of trip purpose. This shows the unique metro choice behaviour of suburban rail travellers in the Indian context.

## Introduction

Modal shift studies aim to assess passenger willingness to shift from one mode to another mode of travel, chiefly in urban regions. Cities in India are introducing metro rail systems to resolve traffic congestion problems. Delhi was the pioneer in construction/operation of metro systems in India, beginning 20 years ago. In later years metro operation was started in Chennai (India) and is under phase-by-phase expansion.

Metro system is one of the attractive urban modes with air-conditioned coaches, and hence a noteworthy shift of passengers is expected from the existing public transport modes such as sub-urban rail. Analysis of modal shift behaviour of different mode users is effectively carried out by developing mathematical models.

In general, modelling studies are conducted in developing countries to understand the shift in behaviour of private mode users [1–6], para-transit users [7, 8], and a few multi-modal users [9] to metro rail systems in order to develop strategies to achieve sustainability. A comparison is made among the selected studies and is presented in Table 1.

**Table 1:** A comparison among the selected studies

S. No.	Country	Shift from	Shift to	Major driver
1	Pakistan	Qingqi	Metro bus system	Travel time, travel distance and cost
2	–	Single-occupant auto	Bus and car pool	Bus convenience, schedule flexibility, cost, safety and a short wait in traffic
3	Malaysia	Private car	Public transport (urban train and bus)	Age, gender, car ownership, travel time, travel cost, household size and income
4	India	Private mode	Metro	Waiting time, travel time and cost
5	India	Bus	Metro	Travel cost (travel time not considered in the study)
6	India	Public transport	Private mode	Age, gender, income
7	India	Para transit	Bus rapid transit system	Travel time
8	China	Auto, taxi, bus, electric bicycle	Metro	Modal transfer facilities
9	Thailand	Private vehicle	Bus rapid transit	Travel time

From the above table, it is evident that travel cost played a major role in determining shift in all studies. However, none of the studies investigated the shift from one rail mode to another, whereas the present study focuses on understanding the shift behaviour of suburban rail users to the proposed metro extension corridor (suburban rail-to-metro rail shift) in Chennai, India. *Since both are rail-based public transport modes and running parallel to each other, the study is useful for both the operators for framing policies to achieve a balanced ridership*.

The study was conducted as part of a feasibility report for the proposed metro rail corridor. To achieve the above objective, a stated preference (SP) questionnaire was prepared consisting of six different proposed scenarios, and the survey was conducted among suburban rail users by a face-to-face interview method. Using the SP data, two modal shift models were developed, one for the entire data and the other for the data pertaining to work trip travellers using the binary logit technique, and probabilities of shift were estimated for both models. The study reveals that the predicted probabilities demonstrate a *unique metro choice behaviour* of suburban rail users.

### Transportation System in Chennai

Chennai, a well-known metropolitan city in India, has an urban population of 4.65 million according to the 2011 Census of India [10]. City transportation is mainly satisfied by road and rail network systems. In particular, the rail system has a vast network consisting of suburban rail, mass rapid transit system (MRTS) and metro rail running across the breadth and width of the city, covering a total length of 356.1 km. The individual route lengths and a line diagram of the three rail systems are presented in Table 2.

**Table 2:** Overview of rail systems in Chennai

S. No.	Rail system	Inception operation	Lines	Total length, km
1.	Suburban rail system	1931	Chennai Central–Arakonam	286
			Chennai Central–Chengalpet	
			Chennai Central–Gummidipundi	
2.	Mass rapid transit system (MRTS)	1997	Chennai Beach–Velachery	25.0
3.	Metro rail system	2015	Washermenpet–Airport*	45.1
			Chennai Central–St. Thomas Mount	

Source: Indian Railways website

*Study corridor under expansion

**Fig. 1:**
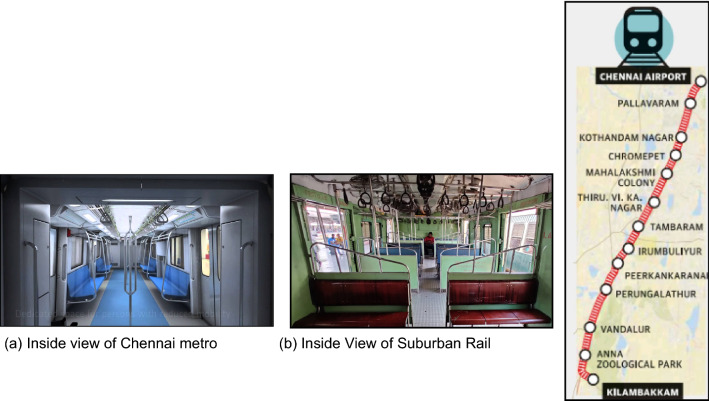
Proposed metro corridor

 The suburban rail system is the oldest and serves commuter needs in three different directions, north, west and south. MRTS is an elevated rail line constructed along Buckingham Canal. Suburban rail and MRTS are operated by the same zonal railways under Indian Railways known as Southern Railways. Both MRTS and suburban rail systems run on broad gauge (1.676 m) and with non-air-conditioned coaches. Metro rail is managed by Chennai Metro Rail Limited (CMRL), the sole authority formed by the Government of Tamil Nadu. Metro started operation with two corridors, one along Anna Salai (also called Grand Southern Trunk [GST] Road) and the other line passing through Poonamallee High Road and Jawaharlal Nehru Road. It runs on standard gauge (1.435 m) and all the coaches are air-conditioned, with large glass windows for a panoramic view. It has partly underground and partly elevated tracks. The present study focuses on the metro corridor along Anna Salai (red line) which runs between Washermenpet and the airport and is under phase II extension (Fig. 1). The extension begins at the airport and ends with Kilambakkam, south of Chennai, planned as an elevated corridor along GST Road.

### Promising Future Transport for Chennai

Metro rail has entered a new era in the transport system in Chennai, with the added advantages compared to the suburban rail system as follows [11]:

- Metro rail has less energy consumption, less space occupancy and carries more passengers.
- Sustainable mode—causes no air pollution, less noise pollution.
- Higher traffic capacity—can haul as much traffic as seven lanes of bus traffic or 24 lanes of car traffic (either way).
- Low ground space occupation—2 m width is sufficient for elevated rail.
- Rapid—reduces travel time by 50% to 75%.
- Metro trains have doors that close before the train starts moving. This should prevent people from hanging outside the trains. Platform screen doors should prevent people from accidentally being pushed onto the tracks while waiting for the trains [12].

However, the metro fare is higher than the suburban rail fare. For instance, metro fare is INR 40 for 10 km, whereas suburban rail fare is only INR 5 for the same distance. This means that a passenger has to pay eight times the fare to travel in the metro. In fact, suburban rail fare is *cheaper than bus fare* in Chennai.

### Competing Corridors: Metro versus Suburban Rail

In accord with the feasibility study (FS) report, the 15.3 km elevated metro line from Chennai Airport to Kilambakkam via Tambaram consists of 13 stations. The proposed corridor is fully elevated, so construction will be easier/cheaper than for underground metro construction. As it is very close to IT hubs, Anna Zoological Park and the proposed Kilambakkam bus stand, the stretch is expected to attract many commuters to the Chennai metro (Fig. 1).

It can be noted that the suburban rail runs almost parallel to the proposed metro corridor. Hence, this will require a changeover for travellers from suburban rail to metro once the corridor is operational. As part of the FS, this study was conducted to understand the possible impact of the metro on suburban rail ridership. The study corridor of suburban rail starts from Chennai Central to Chengalpet via Tambaram and runs south of Chennai. Part of the suburban line, from Tirusulam (Airport) to Urappakkam, runs parallel to the proposed metro extension corridor, leading to competition between the two.

## Literature Review

Studies relevant to the present study are reviewed, and the key observations are given below:

Factors influencing the desires of single-occupant commuters to shift to buses and car pools were studied and policies were recommended to inspire the use of high-occupancy vehicles [1]. The studies found that in buses, convenience was the main factor associated with shift desire. They also found that the thought of car pool comfort does not appear to be important. Aspects like car pool itinerary flexibility, expenditure, security and excessive downtime in traffic were found to be the primary factors linked with potential shift to car pools.

An investigation was conducted in Malaysia to identify factors which had led to an enormous shifting of car riders to the public transport (PT) system [2]. For this purpose, a survey was carried out among the public and car riders, and binary logit models were developed. The study revealed that age, gender, car ownership, travel time, travel cost, household size and income were compelling factors influencing individual choice. Also, it was found that diminished travel time, minimized distance from home to the PT station and promotional fare would boost the usage of public modes.

A modal shift arises when one mode assumes comparative leverage in a travel market over another. The relative advantage can take different forms such as cost, capacity, time, flexibility or reliability. The category of passengers travelling and their circumstances (socio-economic characteristics, purpose of trip, etc.) broadly affect the proportionate importance of each of these factors [13].

The effects of the addition of an exclusive bus lane on auto-rickshaw (three-wheeled motorized para-transit vehicle) users in Chennai was studied using a binary logit model [8]. For this, SP data were collected using the home-interview survey method. A binary logit model of mode choice was developed using the collected data and the model was also validated using the hold-out sample technique. From the above study it was concluded that the shifting of auto-rickshaw users to the exclusive bus lanes varied with respect to time of day (ToD) and level of service (LoS) of the urban road.

The challenges and opportunities in implementing a modal shift from private mode to PT in the UK were analysed in detail [14]. In the real world there were factors like social, political and economic obstacles reducing the expected modal shift. The study concluded that (1) traveller attitudes needed to be recognized, and (2) the quality of the PT system needed to be enhanced to achieve the shift.

A study was carried out to understand the shift behaviour of private transport users and PT users to the existing and proposed metro corridors in Mumbai [3]. For this, a revealed preference survey was conducted among metro users and an SP survey was conducted among the PT users along the proposed metro corridor. A discrete choice model based on revealed preference data showed that 80% of metro users were travelling using PT before shifting to the recent metro line and model based on SP data, which indicated that 60% of private mode users were willing to shift to the proposed metro.

The value of time (VoT) of Alexandria city was estimated by developing a disaggregate linear-in parameter utility-based binary logit mode choice model [15]. The modal attributes (travel time and travel cost) along with traveller attributes (car ownership and income) were chosen as the utility characteristics. Out of 20 models developed, only two models were treated as fruitful in terms of the estimated logical signs and the degree of their significance (*t*-test). The two best models were used to estimate the value of time in Egyptian pounds (LE), with results of 11.30/h and LE 14.50/h, with associated inaccuracy of +3.7% and +33.0%.

Newly introduced metro rail in Chennai has led to growing competition among PT modes [4]. To study the influence of metro rail on bus mode, an SP survey was carried out among express bus passengers. Using the SP data, a modal shift model was developed to estimate the plausible shift from bus to metro rail. Results indicated that factors including fare difference, age and income play an important role in the shift behaviour.

The possibility of shift from the PT system toward private modes such as cars in India due to the unexpected outbreak of COVID-19 was studied [5]. For this, an online questionnaire survey was conducted and logistic regression models were calibrated. The results of model calibration indicated that commuters’ socio-economic attributes such as age, gender and income tend to influence mode shift behaviour. The study concluded that efforts were required to maintain the hygiene of the PT system and that this would restore the confidence of PT users.

Selected studies from the period 1979–2021 pertaining to modal shift were reviewed. It was found that most of the models were developed using a binary logit method. None of the Indian literature studied the impact of metro on suburban rail. The present study considers both travel time and cost saving in an Indian context and tries to predict the probability of shift from suburban rail to metro (rail-to-rail shift). Also, a binary logit model was developed to better understand the modal shift behaviour.

## Modelling Approach

### Selection of Modelling Approach

A disaggregate method was used in the present study. Behavioural disaggregate models have various benefits: minimal data are required for model calibration, and the model is easily transferable and easily understood [16]. From the earlier research pertaining to the subject matter, two approaches were used in disaggregate mode choice analysis: (1) stated preference (SP) approach and (2) revealed preference (RP) approach. The SP approach has many added advantages compared to the RP approach in estimating the choice behaviour of the travellers [17], as follows.

1. Hypothetical, “stated” preferences in controlled experiment set-up
2. Ability to predict reaction to future, not currently existing options
3. Lower cost
4. Accurately specified choice set
5. Numerous answers from each respondent
6. Different choice formats (choices, ranking or rating)
7. Ability to analyse trade-offs among qualitative characteristics

**Fig. 2:**
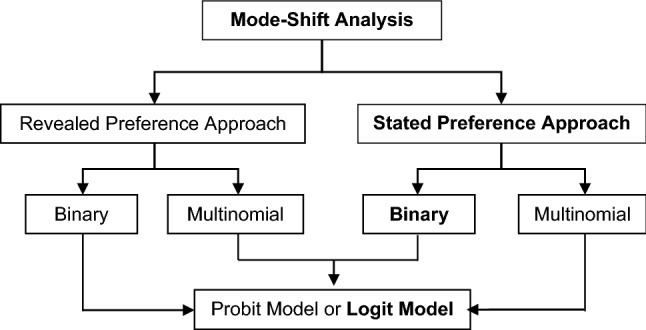
Modelling method for modal shift analysis

Hence, in the present exercise, an SP approach was used for model calibration. Since the modes considered were suburban rail and metro (binary situation), a binary choice model was selected. The interpretation of variables was easier in a logit model than a probit model, and hence the logit model was used for the study. Figure 2 shows the modelling approach, and the bolded letters of the flowchart indicate the sequence of steps related to the model calibration in the present study.

### Preparation of SP Questionnaire

Stated preference methods try to determine people's willingness to pay by directly asking them how much they value certain environmental goods or services through an appropriately designed survey questionnaire [18]. SP surveys are most widely used in transportation studies to reveal how changes to infrastructure or services will alter travel shift behaviour. These are SP survey questionnaires that desire to gather data on the stated behaviour of mode users towards hypothetical stated contexts [19]. An SP questionnaire was prepared to understand commuter shift behaviour. The survey was conducted among suburban rail users (272 samples) considering different trip purposes. The factors that have a significant influence on the choice of metro can be divided into three general categories as follows:

- *Socio-economic characteristics*: gender; age of the commuters; monthly family income in Indian rupees (INR: 1 euro ≈ 81 INR); vehicle ownership; place of work; frequency of travel; educational level; employment sector
- *Details on current trip*: purpose of trip; mode and stages of travel; trip length
- *Stated hypothetical scenarios*: (six scenarios as given below)
  - Scenario (1): travel time saving is 25%: 2 times the MTC bus fare
  - Scenario (2): travel time saving is 25%: 3 times the MTC bus fare
  - Scenario (3): travel time saving is 25%: 4 times the MTC bus fare
  - Scenario (4): travel time saving is 50%: 2 times the MTC bus fare
  - Scenario (5): travel time saving is 50%: 3 times the MTC bus fare
  - Scenario (6): travel time saving is 50%: 4 times the MTC bus fare

## Model Development

The model developed is a binary logit model. Binary models serve a number of functions. First, the ease of binary choice settings makes it possible to develop a range of practical models which gives greater flexibility in more complicated choice situations. Second, there are many basic theoretical problems that are simple to illustrate in the context of binary choice. Since our aim is to study the shift behaviour of suburban rail travellers to proposed metro rail, only two modes were considered (suburban rail and metro; binary in nature). The structure of the model is as follows:

$$P_{{{\text{shift}}}} = \frac{{\exp \left( {a_{0} + a_{1} X_{1} + a_{2} X_{2} + ... + a{}_{n}X{}_{n}} \right)}}{{1 + \exp \left( {a_{0} + a_{1} X_{1} + a_{2} X_{2} + ... + a{}_{n}X{}_{n}} \right)}}$$

where *P*_shift_ is the probability of shift from suburban rail to metro, *X*_1_, *X*_2_, *X*_3_ ... are factors influencing change behaviour (factors include socio-economic and travel characteristics of suburban rail passengers), and a_0_, a_1_, a_2_ ... are model attributes to be calibrated using the model development exercise.

### Coefficient of Correlation (*r*)

In statistics, correlation is a way of establishing the relationship/association between two variables (x,y). In other words, the correlation coefficient formula helps in calculating the correlation coefficient which measures the dependence of one variable on the other variable. Correlation is measured numerically using the following formula:

$$r = \frac{{\sum {\left( {x_{i} - \overline{x} } \right)\left( {y_{i} - \overline{y} } \right)} }}{{\sqrt {\sum {\left( {x_{i} - \overline{x} } \right)^{2} \sum {\left( {y_{i} - \overline{y} } \right)^{2} } } } }}$$

where *x*_i_, *y*_i_ = variable value, *x*, *y* = mean values

Before beginning the modelling exercise, it is necessary to identify the significant influencing variables in explaining the shift behaviour. For this, the correlation coefficient can be used as a tool in ranking the variables. A correlation matrix is developed which includes all the independent variables (*X*_1_, *X*_2_, *X*_3_ ...) and the dependent variable (*P*_shift_). Table 3 presents the correlation levels, and based on this matrix, certain conclusions are drawn.

**Table 3:** Correlation matrix

	1	2	3	4	5	*6*	7	8	9	10	11	12	13	14	15	16	17	18	19	20	21	22	23	24	25	26	27
1	-	0.04	− 0.09	0.05	0.07	− 0.07	0.03	-	0.07	0.01	− 0.06	− 0.07	0.01	0.04	0.09	− 0.12	0.12	− 0.11	0.04	0.10	0.19	− 0.07	0.18	0.16	0	0	− 0.08
2		-	− *0.60*	− 0.19	− 0.06	− 0.04	− 0.03	-	0.01	− 0.02	− 0.15	− 0.16	− 0.16	− 0.08	− 0.08	− 0.16	0.16	− *0.50*	*0.58*	0.09	0.08	− 0.10	0.04	− 0.03	0	0	0.03
3			-	− *0.62*	− 0.19	− 0.03	0.04	-	− 0.05	0.02	0.22	0.18	0.08	0.01	− 0.07	0.05	− 0.05	0.17	− 0.28	0.05	− 0.02	0.10	− 0.04	0.04	0	0	0.05
4				-	− 0.06	0.08	− 0.03	-	0.07	− 0.07	− 0.11	− 0.05	0.08	0.07	0.18	0.10	− 0.10	0.26	− 0.18	− 0.14	− 0.06	− 0.02	− 0.01	− 0.02	0	0	− 0.08
5					-	− 0.01	− 0.01	-	− 0.02	0.19	− 0.07	− 0.09	− 0.05	− 0.03	− 0.03	− 0.02	0.02	0.05	− 0.06	− 0.01	0	− 0.02	0.07	0	0	0	− 0.05
6						-	− 0.01	-	− 0.01	− 0.02	− 0.04	− 0.06	− 0.03	− 0.02	− 0.02	0.04	− 0.04	0.07	− 0.04	− 0.05	− 0.03	− 0.05	− 0.04	− 0.04	0	0	− 0.02
7							−	−	− 0.01	− 0.02	− 0.03	− 0.04	− 0.02	− 0.01	− 0.01	0.03	− 0.03	0.05	− 0.03	− 0.03	− 0.02	− 0.03	− 0.01	0.02	0	0	− 0.01
8								−	-	-	-	-	-	-	-	-	-	-	-	-	-	-	-	-	-	-	-
9									-	-0.04	-0.07	-0.10	-0.06	-0.03	-0.03	-0.07	0.07	0.01	-0.06	0.04	0.09	0.10	0.25	0.28	0	0	-0.02
10										-	-0.13	-0.17	-0.10	-0.05	-0.05	0.03	-0.03	0.01	-0.02	0	0.05	0.13	0.18	0.15	0	0	-0.01
11											-	-0.32	-0.18	-0.09	-0.09	-0.03	0.03	0.07	-0.15	0.05	-0.04	-0.02	0.01	0.02	0	0	0.06
12												-	-0.25	-0.13	-0.13	0.08	-0.08	0.17	-0.23	-0.01	-0.03	-0.04	-0.06	-0.03	0	0	0.02
13													-	-0.07	-0.07	0.07	-0.07	0.13	-0.13	-0.05	-0.02	0.01	-0.10	-0.07	0	0	-0.05
14														-	-0.04	-0.02	0.02	0.03	-0.08	0.03	-0.08	-0.01	-0.06	-0.06	0	0	-0.04
15															-	-0.08	0.08	0.11	-0.08	-0.06	-0.08	0	-0.02	-0.04	0	0	0.04
16																-	-1.00	0.43	0.09	-0.57	0.05	-0.27	-0.27	-0.24	0	0	-0.06
17																	-	-0.43	-0.09	0.57	-0.05	0.27	0.27	0.24	0	0	0.06
18																		-	-0.54	-0.70	0.02	-0.08	-0.10	-0.01	0	0	-0.02
19																			-	-0.23	0.02	-0.08	-0.09	-0.14	0	0	0
20																				-	-0.04	0.17	0.19	0.14	0	0	0.02
21																					-	-0.16	0.34	0.26	0	0	0
22																						-	0.36	0.38	0	0	0.01
23																							-	0.82	0	0	-0.04
24																								-	0	0	-0.03
25																									-	0	-0.02
26																										-	-0.65
27																											-

Code indications:

**Table:** 

1	Gender of the passenger	8	Income of passenger 5001–7500	15	Income of passenger >50,000	22	Travel cost, INR
2	Age of passenger <20	9	Income of passenger 7501–10,000	16	Daily trip maker	23	Travel time, min
3	Age of passenger 21–40	10	Income of passenger 10,001–15,000	17	Occasional trip maker	24	Travel distance, km
4	Age of passenger 41–60	11	Income of passenger 15,501–20,000	18	Work trip purpose	25	Travel time saving
5	Age of passenger >60	12	Income of passenger 20,001–30,000	19	Educational trip purpose	26	Fare difference
6	Income of the passenger <2000	13	Income of passenger 30,001–40,000	20	Other trip purpose	27	Probability of shift
7	Income of the passenger 2001–5000	14	Income of passenger 40,001–50,000	21	Number of stages		

### Choice of Variables

From the correlation matrix, the following variables are chosen for model development based on the ranking of correlation coefficients from maximum to minimum. Variables have a correlation coefficient value with the probability of shift (dependent variable) equal to or greater than ±0.05 and are selected and listed in Table 4.

**Table 4:** Selected variables for model development

S. No.	Variable	Correlation with *P*_shift_
1	Fare difference	−0.65
2	Gender	−0.08
3	Passenger age 41–60	−0.08
4	Daily trip makers	−0.06
5	Passenger age >60	−0.05
6	Passenger income 30,001–40,000	−0.05
7	Passenger age 41–60	0.05
8	Passenger income 15,001–20,000	0.06
9	Occasional trip makers	0.06

### Inter-correlation Between Selected Variables

Once again a correlation matrix is developed from the previous correlation matrix consisting of only ten selected variables (including the dependent variable) to determine whether any inter-correlation exists among the selected variables. If inter-correlation exists, such a pair of variables can be isolated and any one among them selected based on the correlation with the dependent variable. Thus the developed inter-correlation matrix is presented in Table 5.

**Table 5:** Inter-correlation matrix

	1	3	4	5	11	13	16	17	26	27
1	–	−0.09	0.05	0.07	−0.06	0.01	−0.12	0.12	0	−0.08
3		–	*−0.62*	−0.19	0.22	0.08	0.05	−0.05	0	0.05
4			–	−0.06	−0.11	0.08	0.10	−0.10	0	−0.08
5				–	−0.07	−0.05	−0.02	0.02	0	−0.05
11					–	−0.18	−0.03	0.03	0	0.06
13						–	0.07	−0.07	0	−0.05
16							–	−*1.00*	0	−0.06
17								–	0	0.06
26									–	−0.65
27										–

## Results analysis

1. Daily trip makers were highly correlated with occasional trip makers (1.0). The correlation with the dependent variable was also equal with opposite signs. However, if the sample percentage was considered, daily trip makers were significant in the survey output (84.5%). Hence, the variable daily trip maker was included in the model.
2. Similar to the above case, three age ranges influenced the probability of shift. If the correlation coefficient was considered, all three variables were intercorrelated, and passengers with age range of 41–60 influenced the dependent variable in a better way compared to the other two. However, if the sample percentage was considered, passengers with age range of 21–40 were significant in the survey output (66.9%). Hence, the variable passenger age range 21–40 was included in the model.
3. Considering income ranges, two income ranges 15,001–20,000 and 30,001–40,000 influenced the dependent variable. Comparing both variables in terms of correlation value and sample size, the income range 15,001–20,000 (0.06 and 19.1%) was included in the model development.
4. From the above discussion, five variables were finally chosen for model development: (a) fare difference, (b) gender, (c) daily trip makers, (d) passengers with age range 21–40 and (e) passenger income range 15,001–20,000.

### Full Model

Full model development is done step-by-step as discussed below:

- **Step 1:** *Model type*: For estimating the probability of shift from suburban rail to the proposed metro line, a binary logit model is developed using SP data. The collected SP data are coded in Microsoft Excel using binary codes (1, 0).
- **Step 2:** *Model development*: The model is developed considering only the chosen variables based on the correlation coefficient values (*r*). The total number of suburban rail passengers interviewed is 272 (sample size) and opinions are collected for six different *fare difference* in combination with *travel time saving* scenarios. Hence, the total number of data points used in the model is 1672 (i.e. 272×6).
- **Step 3:** *Model validation*: Among 1672 data points, 1331 are used for model development. The remaining 301 data points are kept as the *hold-out sample* for model validation purposes. The result of model development using 1331 data points is given in Table 6 below:

**Table 6:** Model development (using 1331 data points)

Variable	Estimated coefficient	*t* statistic
Constant	7.347	15.19
Gender	−0.739	−3.85
Age 21–40	0.387	2.34
Income 15,001–20,000	0.467	2.45
Daily trip makers	−0.847	−4.12
Fare difference	−2.271	−18.82
Initial log-likelihood	−922.58	
Final log-likelihood	−562.18	
ρ^2^	*0.39*	

Thus the developed model is applied to the hold-out sample of 301 data points and the log-likelihood (LL) values are calculated by applying the above model on the hold-out sample and compared with the software-estimated LL as shown in Table 7. It can be observed that the two LL values are reasonably close, and hence the developed binary logit model is validated.

**Table 7:** Model validation

	Data set (1): 1331 nos.	Data set (2): 301 nos.
Initial LL	−922.58	−208.64
Final LL	−562.18	−126.44
ρ^2^	*0.39*	0.39
Estimated LL	−126.44	
Calculated LL using the model	−107.63

- **Step 4:** *Recalibration of model*: The model is recalibrated once again with 1632 data points for model application and the developed model is presented in Table 8 below.

**Table 8:** Recalibrated model (using 1632 data points)

Variable	Estimated coefficient	*t* stat.
Constant	7.450	17.52
Gender	−0.788	−4.45
Age 21–40	0.317	2.14
Income 15,001–20,000	0.413	2.34
Daily trip makers	−0.744	−3.94
Fare difference	−2.315	−23.66
Initial log-likelihood	−1131.2	
Final log-likelihood	−694.31	
ρ^2^	*0.39*

*Positive observation:* From the recalibrated model, it can be observed that suburban rail travellers falling in the age group of 21–40 (with estimated coefficient = +0.317) and monthly income of 15,001–20,000 (with estimated coefficient = +0.413) are more willing to use metro once it started operation in the study corridor.

### Probability of Shift (Full Model)

Using the recalibrated model, the probability of shift from suburban rail to metro is estimated for the proposed fare and travel time saving scenarios and is presented in Table 9.

**Table 9:** Probability of shift to metro rail

Scenario	% Shift
Case i: Travel time saving = 25%	
Metro fare is 2 times the bus fare	85.4
Metro fare is 3 times the bus fare	38.8
Metro fare is 4 times the bus fare	6.4
Case ii: Travel time saving = 50%	
Metro fare is 2 times the bus fare	85.4
Metro fare is 3 times the bus fare	38.8
Metro fare is 4 times the bus fare	6.4

From the above table, it is evident that the travel time saving does not have a significant impact on the shift of passengers from suburban rail to metro rail. The reason may be that suburban rail travellers feel that the metro does not save travel time when compared to their current mode of travel.

It is also observed that when the metro fare is just two times the bus fare, then 85.4% of suburban passengers show interest in the proposed metro, whereas if the fare is three and four times the bus fare, then the shift is only 38.8% and 6.4%, respectively. This means that the suburban travellers are sensitive to fare changes.

### Work Trip Model

Most urban travel demand analysis models were structured for different trip purposes separately. In other words, it was usually assumed that the demand for different activities, such as work, recreation and so forth, were independent [20]. Hence the SP questionnaire includes a question on trip purpose—work, education or other trip purpose. A comparison of the share of each trip purpose reveals that 62.5% of suburban travellers were making a work trip (i.e. daily trip makers). Hence, it is interesting to develop a model to understand how work trip makers shift behaviour. A sub-model was developed for data consisting only of work trip makers and the results are given in Table 10.

**Table 10:** Model development and probability of shift for work trip makers (using 1020 data points)

Variable	Estimated coefficient	*t* statistic
Constant	7.137	10.64
Gender	−0.403	−1.95
Age 21–40	0.603	3.05
Income 15,001–20,000	0.538	2.55
Daily trip makers	−1.201	−2.6
Fare difference	−2.243	−16.98
Initial log-likelihood	−707.01	
Final log-likelihood	−441.91	
ρ^2^	*0.37*	

*Scenario*	*% Shift*
Case i: Travel time saving = 25%	
Metro fare is 2 times the bus fare	83.6
Metro fare is 3 times the bus fare	38
Metro fare is 4 times the bus fare	6.6
Case ii: Travel time saving = 50%	
Metro fare is 2 times the bus fare	83.6
Metro fare is 3 times the bus fare	38
Metro fare is 4 times the bus fare	6.6

*Negative observation:* From the above work trip model, it can be observed that suburban male passengers (with estimated coefficient = −0.403) and daily trip makers (especially work/education trip purpose) (with estimated coefficient = −1.201) are not willing to use the proposed metro.

## Conclusions

The present study aimed to study the shifting behaviour of suburban rail passengers towards the newly proposed metro extension corridor in an Indian context. To accomplish this, an SP questionnaire survey was conducted among suburban rail travellers and a binary logit model (suburban rail and metro) was calibrated to estimate the probability of shift to metro. From the developed models, it is understood that suburban rail passengers assigned less importance to the travel time saving, while fare difference played a major role in the probability of shift, irrespective of trip purpose. This shows the unique propensity of urban rail travellers in the Indian context. The major findings of the study are as follows:

1. From the correlation value (*r*), it is clear that suburban rail travellers falling into the age groups of less than 20 (*r* = +0.03) and 21–40 (*r* = +0.05) are willing to shift to metro in the study. This means that the younger group of travellers shows more interest in metro than older people. It is true from the model output that suburban rail passengers falling in the age group of 21–40 (+0.317/ *t* stat: +2.14), with monthly income 15,001–20,000 (+0.413/ *t* stat: +2.34) are more willing to use metro, while daily travellers are reluctant to use metro (−0.744/ *t* stat: −3.94).
2. From the correlation value, it is observed that the suburban travellers with age greater than 60 fall in the income range of INR 10,000–15,000 (*r* = +0.19). Since the metro fare is comparatively higher than the suburban rail fare, senior people are not willing to shift to metro. Table 11 compares the metro fare with the suburban fare.

**Table 11:** Comparison between the metro fare with the suburban fare

Criterion	Metro fare (INR)	Suburban rail fare (INR)
Minimum fare	10	5
Maximum fare	50	30
Maximum distance	40 km	155 km
Per km fare	1.25	0.19

(3) Fare difference (*r* = −0.65) plays a significant role in determining shift behaviour of suburban rail travellers.
(4) The correlation between number of stages and travel cost is significantly high, with a negative sign (*r* = −0.16). This indicates that in order to reduce travel cost, travellers use different modes in a single trip.
(5) Compared to daily travellers (*r* = −0.06), occasional travellers (*r* = +0.06) are more likely to use the metro service.
(6) Travel time and travel distance are highly positively correlated (*r* = +0.82), as expected.
(7) Compared to female travellers, male travellers are less willing to use metro (−0.788/ *t* stat: −4.45).
(8) Similar behaviour to the above is observed for work trip travellers.
(9) From the probability of shift (Table 10), it is clear that the probability of shift is highly sensitive to metro fare change. For example, the shift is from 84% to 38% for a fare change from two to three times that of bus fare.

The future work and suggestions are as follows.

- The current study considered cost and travel time saving in stated scenarios. In future studies, other sustainable modes such as walking, cycling and cycle rickshaw as feeder service choice can be investigated for model development.
- One of the aspects of PT service is travel time reliability (TTR) [21]. From the study, it is understood that the commuters are unaware of the importance of travel time saving by using metro rail. CMRL must conduct a study on the TTR of metro trains and take steps to promote awareness of this benefit among the public using the study results.

## Data Availability

The majority of data used in this study were collected through primary surveys.
